# The Need for Inspiration and Admiration

**DOI:** 10.1093/function/zqae001

**Published:** 2024-01-03

**Authors:** Ole H Petersen

**Affiliations:** School of Biosciences, Sir Martin Evans Building, Cardiff University, Cardiff CF10 3AX, UK

**Keywords:** creativity, admiration, inspiration, Nobel Laureates, talented students

The German writer and Nobel Prize winner, Thomas Mann, repeatedly expressed the view that the gift of admiration is indispensable for creativity. I believe that this is not only the case in the arts, but also the case in science. Fundamentally, this is the reason for the almost universal system of prizes and other rewards, which is an important element in what we may call the scientific world. By recognizing and signposting great achievements, we provide examples worthy of emulation. It does not matter whether the object of admiration appreciates the recognition. An Australian literary critic wrote about the Australian writer and Nobel Prize winner, Patrick White, that we (Australians) put him on a pedestal, and he complained about the view, but who else did we have? The fact that the French writer and philosopher Jean-Paul Satre declined the Nobel Prize for literature made no difference to the signposting of his excellence; if anything, it probably enhanced his reputation.

Given that the worlds of the arts and the sciences are enormous and that it is therefore impossible for anyone to know everything that has been created, we need some system for selecting the most valuable elements. Currently, in the sciences, this often seems to be done by citation analysis. We count the number of citations to papers in a journal in relation to the number of papers published (Impact Factor), thereby thinking that we can determine the quality of the journal. We count citations to individual papers or assess individual lifetime achievements by calculating the h-index. Clearly, none of these metrics measure quality, but just interest in the work published, and if one were to look at this from the perspective of the arts, one might be very surprised. Do we, for example, judge the quality of a film on the basis of the number of reviews it has received? Or do we judge the quality of a book by counting the number of copies sold?

The classical, and still continuing, system of recognizing excellence in the sciences is by the award of prizes, most famously the Nobel Prizes, and by competitive elections to science academies, for example, the Royal Society, the National Academy of Sciences, and the German National Academy of Sciences Leopoldina, to name just a few of the most well-established and visible academies. Like all human endeavors, the judgements made are not always right. Mostly, those selected deserve their awards, but there is always the problem of the borderline cases.

For the professionals in the various branches of the sciences, the reward system is clear, and it is not difficult to know who has received recognition. However, those who might want to pursue a scientific career may not have easy access to this information. Many school leavers know very little about those working in the sciences and therefore fail to be inspired to join this important branch of human creative activity. In my experience, most school leavers with an interest in biological sciences want to study medicine, but do not seriously consider a scientific career unless they fail to get into medical school. This is, obviously, not a happy situation for the sciences.

Hungary seems to have found a partial solution to this problem by the creation of the National Academy of Scientist Education (https://www.edu-sci.org/szeged-scientists-academy/mission/?lang=en). It is the brainchild of Peter Hegyi, Professor of Gastroenterology at Semmelweis University in Budapest, Member of Academia Europea, and member of *FUNCTION*’s editorial board. Peter is the Programme Director and he managed to persuade the Nobel Laureate Bert Sakmann to accept the position as Director General of Education. The general objective of the program is to retain the most talented students of the country in Hungary, and to establish a center that would motivate talented foreign young people to settle down in Hungary. The academy aims to guide talented students through the steps of becoming scientists from their secondary school years. There are 7 education centers distributed across Hungary and these centers are led by scientists who are known as Szent-Györgyi (named after the Hungarian Nobel Laureate, Albert Szent-György) Senior Teachers. It is their job to find and mentor the pupils within their region who are particularly interested in natural sciences. When the school leavers enter university, they receive further research training in 1 of 4 cities (Budapest, Debrecen, Pecs, or Szeged) at the best equipped laboratories in the country under guidance of Szent-György Mentors. The students will thus start research work along with their university studies. In Szeged, where the program started, the students may stay at the Bert Sakmann Dormitory, which was opened in the spring of 2022. Bert Sakmann donated the original duplicate of his Nobel Prize Medal and Diploma to the Academy and it is on display in the foyer of the college.

The highlights of the Academy’s program are the 2 annual gatherings in Szeged of what is known as the meetings of Nobel Laureates and Talented Students. In December 2023, I was privileged to be 1 of the 4 principal guest speakers (Figure 1) in the 21st Meeting of this kind (https://www.edu-sci.org/konferencia202312/index_eng.html). On this occasion, more than 500 pupils from secondary schools as well as University students from all over Hungary came to the conference and participated in a varied program of presentations by students and mentors as well as roundtable discussions with the 4 Guest Lecturers, culminating in an afternoon of Plenary Lectures by the Nobel Laureates Randy Schekmann (UC Berkeley) and Tom Südhof (Stanford University) as well as Miklos Sahin-Toth (UCLA), and myself (Cardiff University). Billed as a “Celebration of Excellence,” it commemorated the 10th Anniversary of the Nobel Prize Awards to Schekmann and Südhof, as well as the 60th and 80th birthdays, respectively, of Sahin-Toth and myself.

**Figure 1.**
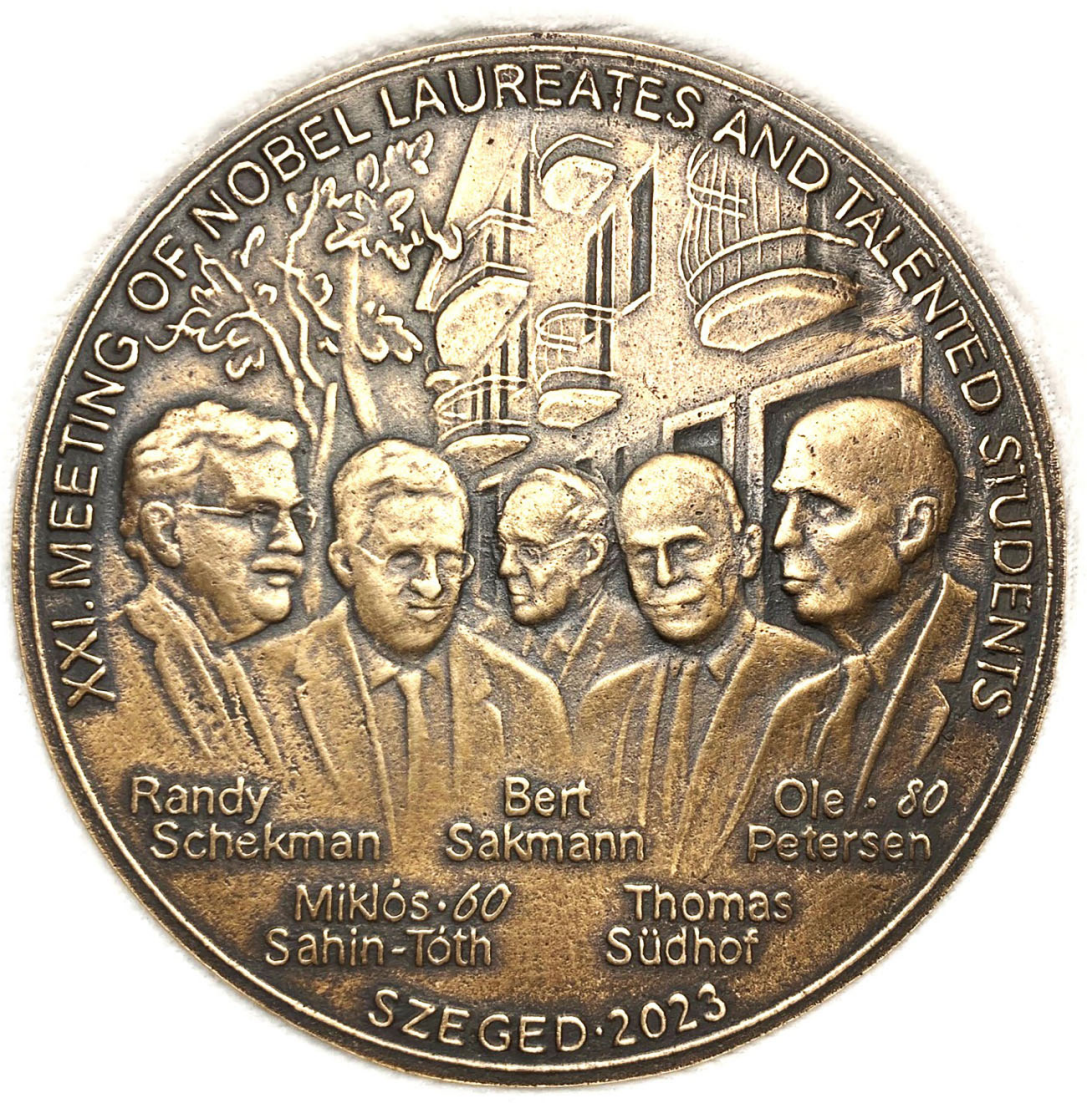
Picture of the medal commemorating the 21st Meeting of Nobel Laureates and Talented Students at the University of Szeged, Hungary, December 2023.

The interactions with the school leavers and university students were very valuable, and at the final press conference, held at Szeged’s Town Hall, all 4 of us commented very positively on the extremely high quality of the school pupils and University students. The prolonged roundtable discussions had strayed into many different areas, partly scientific and partly more general, both philosophical and political. What was impressive was the seriousness of the students and their willingness, and ability, to engage in debates. In the interactions with me, they were particularly keen to learn about the key decisions I had made in my career concerning research themes and places of work. Another important theme was the state of Hungary, and the students were keen to know my opinion. As far as I can see, Hungary has a good school and university education system, better than many other European countries. Hungarian scientists have also made enormous contributions to our scientific knowledge, although many have done so working outside the country. On the other hand, Hungarian politics have been consistently bad, and this clever country has repeatedly found itself on the wrong side of history. Sadly, the bad political choices do not seem to have come to an end. Nevertheless, I left Hungary hopeful, mainly because of the very positive attitudes of the school leavers and university students. They will be the thought leaders of the future and will hopefully have been inspired by the encounter with top scientists early in their lives. It would seem a good idea for many other countries to create schemes along the lines of those established by the Hungarian National Academy of Scientist Education.

## Data Availability

There are no data in this editorial.

